# Induction of Salt Stress Tolerance in Wheat Seeds by Parental Treatment with Salicylic Acid

**DOI:** 10.3390/plants13233373

**Published:** 2024-11-30

**Authors:** Lei Yan, Xue Jiang, Yuman Zhang, Yongwen Dong, Can Zhao, Ke Xu, Zhongyang Huo, Weiling Wang

**Affiliations:** Jiangsu Key Laboratory of Crop Cultivation and Physiology, Jiangsu Co-Innovation Center for Modern Production Technology of Grain Crops, Yangzhou University, No. 88 Daxue South Road, Yangzhou 225009, China; mz120231385@stu.yzu.edu.cn (L.Y.); mz120231361@stu.yzu.edu.cn (X.J.); mx120240739@stu.yzu.edu.cn (Y.Z.); mz120231414@stu.yzu.edu.cn (Y.D.); canzhao@yzu.edu.cn (C.Z.); xuke@yzu.edu.cn (K.X.)

**Keywords:** salicylic acid, salt tolerance, germination, offspring, physiological mechanism

## Abstract

Soil salinization is an important factor that limits crop production. The effects of spraying salicylic acid (SA) during the grain-filling stage on the salt tolerance of progeny seeds in wheat (*Triticum aestivum* L.) were investigated in this study. The results showed that spraying SA during the grain-filling stage significantly increased the grain weight and yield of wheat plants. Meanwhile, the seeds from the SA-treated plants showed a higher germination rate, length and dry mass of the coleoptile and radicle, and a lower mean germination time compared to the seeds of water-treated plants under the salt germination condition, indicating that SA pretreatment during the grain-filling stage could effectively improve the salt tolerance of progeny seeds in wheat. SA pretreatment significantly increased the activities of amylases and the respiration rate, accompanied by a decrease in starch content, and a higher accumulation in the level of soluble sugars and adenosine triphosphate (ATP) in the germinated seedlings compared to the water pretreatment under salt stress. In addition, SA pretreatment obviously alleviated the increase in malondialdehyde (MDA) content and the reactive oxygen species (ROS) release rate in seedlings by activating antioxidant enzymes (superoxide dismutase (SOD) and peroxidase (POD)) under salt stress. Moreover, the seedlings of the SA-treated plants showed lower Na^+^ and higher K^+^ contents compared to the seeds of water-treated plants under salt stress. The results of this study indicate that spraying SA during the grain-filling stage improves the capacity of offspring seeds to maintain osmotic and ion balance and redox homeostasis under salt stress, thereby conferring salt tolerance to the wheat seeds.

## 1. Introduction

With the growth in population and the expansion of urbanization, global food demand is projected to increase by 35% to 56% by 2050 compared to 2010 [1]. Under the influence of global climate change, improper fertilizer application, unreasonable irrigation methods, and other factors have caused more than one-third of the world’s soil to be affected by salinization and alkalinization [2]. Saline-alkaline soils impose multiple adverse effects on plants, mainly including osmotic stress and ion toxicity, severely inhibiting plant growth and development [3,4]. As a result, the efficiency of food production on saline-alkaline soils is significantly lower compared to normal soils [5]. Therefore, improving the salt tolerance of crops to enhance the productivity of saline-alkaline soils is an important strategy for addressing global food security challenges.

Seed germination is a critical stage in plant morphological establishment, but it is particularly sensitive to salt stress [6]. Salt stress has a direct and severe impact on seed germination. This is primarily manifested by the low water potential of saline soil solutions, which inhibits the ability of the seed to imbibe water [7]. This inhibition hampers the radical and plumule from breaking through the seed coat, thereby prolonging the germination time [8]. In addition, salt stress increases the content of abscisic acid (ABA) while decreasing the level of gibberellin (GA), which leads to the reduction in amylase activity in seeds, resulting in slow degradation of the endosperm starch and inhibited seed germination [9,10]. Moreover, the excessive accumulation of sodium (Na^+^) leads to the buildup of reactive oxygen species (ROS) in seeds under salt stress, causing oxidative damage of the cell membrane, protein, and nucleic acid [11,12]. Therefore, enhancing the germination ability of crops under salt stress conditions can contribute to the growth of more robust seedlings, ultimately improving the overall crop yield and quality.

It has been documented that plants possess a transgenerational memory of environmental stresses, which is an important mechanism for plant offspring to acquire stress tolerance [13]. For example, the activity of antioxidant enzymes in plants under drought conditions can be effectively inherited by their offspring in wheat plants [14]. The progeny of plants subjected to waterlogging stress exhibited a higher net photosynthetic rate, antioxidant enzyme activity, and biomass than the progeny of plants that had not experienced waterlogging stress [15]. The seeds collected from plants cultivated under terminal drought stress showed a stronger salt tolerance compared to the seeds collected from plants under well-irrigated conditions [13]. Previous studies have found that epigenetic variations (such as DNA methylation, histone modifications, or non-coding RNAs) play a crucial role in transgenerational inheritance by mediating responses across generational layers to cope with environmental stresses [16]. It has been proven that salicylic acid (SA) is associated with epigenetic modifications to mediate bacterial infection-induced stress tolerance in the next generation.

Salicylic acid is an endogenous physiological regulator that belongs to the class of phenolic compounds, which plays an important role in the plants’ responses to both biotic and abiotic stress including salt stress [17,18]. It has been reported that an appropriate dose of exogenous SA can alleviate the adverse effects of salt stress on seed germination in many crops [19]. For example, exogenous SA can alleviate ion toxicity by reducing the Na^+^ content, thereby lowering the Na+/K+ ratio, which helps maintain ROS and hormone homeostasis. This process promotes starch hydrolysis, providing sufficient energy for seed germination, ultimately improving rice seed germination under salt stress [20]. Previous research has shown that exogenous SA alleviates salt stress damage in ryegrass by optimizing photosynthesis and antioxidant metabolism [21]. Exogenous SA also promoted the germination of *Limonium bicolor* (Kuntze) seeds by enhancing the activity of amylase [22]. However, there is limited research on the impact of exogenous SA application on the stress tolerance of subsequent generations, especially in food crops.

Wheat is a globally important staple crop, and is also known as the staple food for nearly 35 per cent of the world’s population [23]. The grain-filling stage is a critical period for wheat grain development [24]. A previous study showed that spraying an appropriate concentration of SA solution during the grain-filling stage significantly increased the wheat yield and improved the grain quality [25]. However, the effects of spraying SA during this critical period on the salt tolerance of its progeny seeds remain unclear. In the present study, it was hypothesized that the SA-treated wheat plants could induce salt stress tolerance in their progeny. The effects of SA trans-generation priming were analyzed, and its underlying mechanisms were discussed in this study. The findings of this study are expected to contribute to the development of practical strategies for improving salt tolerance in wheat during the early stages of plant growth.

## 2. Materials and Methods

### 2.1. Chemical Treatments and Seed Collection

**Figure 1:**
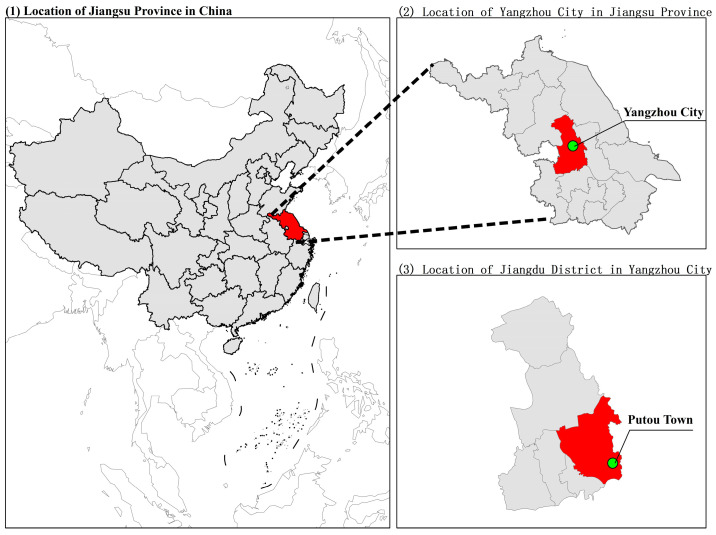
Location of Putou Town, Jiangdu District, Yangzhou City, Jiangsu Province, China.

This experiment was conducted from 2021 to 2022 in Putou Town, Jiangdu District, Yangzhou City, Eastern China (geographical coordinates: 119.44° E, 32.43° N, with an elevation of approximately 3.5 m) (Figure 1). The location is situated on the lower reaches of the Yangtze River Plain, with an average annual temperature of about 15.7 °C and an annual precipitation of around 1000 mm. The wheat variety used in the experiment was Yangmai 23 (*Triticum aestivum* L.), a variety widely planted locally. The experiment was carried out using manual row sowing with a row spacing of 25 cm and a basic seedling density of 2.4 million plants per hectare. The sowing date was 9 November 2021. A single application of phosphorus and potassium fertilizers was used as the base application, and the application ratio of nitrogen to phosphorus and potassium as 2:1:1. Fertilization included the application of 240 kg of nitrogen (N) per hectare, and 120 kg each of phosphorus (P_2_O_5_) and potassium (K_2_O) per hectare.

Five days after the flowering of Yangmai 23 (in 20 April 2022), foliar spraying was applied to the wheat plants. Four different concentrations of SA solutions (prepared from Sigma-Aldrich Co., Ltd., St. Louis, MO, USA) were used in the experiment: 0, 50, 150, and 450 μmol/L. Spraying was conducted once every seven days for a total of three applications, with each spraying carried out at dusk. The volume of each application was 450 L per hectare, and the solution contained 0.1% Tween 20. Each treatment was repeated three times, with each replicate covering an area of 12 square meters. After the wheat matured, the grains were harvested (1 June 2022), and the thousand-grain weight and yield were measured.

### 2.2. Salt Stress Treatment During Seed Germination

The seeds collected from the pretreated wheat plants were surface sterilized with 10% hydrogen peroxide for 10 min and then thoroughly rinsed with distilled water. Fifty seeds pretreated with varying concentrations of salicylic acid (0, 50, 150, and 450 μmol/L) were placed evenly onto Petri dishes containing two layers of filter paper. To simulate salt stress, 10 mL of a 100 mmol/L sodium chloride (NaCl) solution was added to each dish. Seeds pretreated with water and germinated under normal conditions were used as the control group. Finally, the following five treatments were established: WW (water pretreatment + distilled water germination), WS (water pretreatment + salt treatment), S_50_S (50 µmol/L SA pretreatment + salt treatment), S_150_S (150 µmol/L pretreatment + salt treatment), and S_450_S (450 µmol/L SA pretreatment + salt treatment). All dishes were incubated at 20 ± 0.5 °C for 7 days in an incubator under darkness; each treatment was repeated 3 times. The germination rate, mean germination time, length of the radicle and coleoptile, and dry weight of the radicle, coleoptile, and seed residue in each treatment were investigated to evaluate the effects of the exogenous application of SA during the grain-filling stage on the salt tolerance of offspring in wheat.

To explore the mechanisms of the SA-induced transgenerational salt tolerance in wheat, we designed four treatments: WW (water pretreatment + distilled water germination), WS (water pretreatment + NaCl treatment), SW (150 µmol/L SA pretreatment + distilled water germination), and SS (150 µmol/L SA pretreatment + NaCl treatment), and the seed samples were collected at 24, 48, and 72 hours after salt treatment for the biochemical and physiological measurements; each treatment was replicated in 20 Petri dishes. The seed surface sterilization, NaCl concentration, and germination conditions were consistent with those above.

### 2.3. Grain Yield and 1000-Kernel Weight

At maturity, 1 m^2^ wheat plants from each treatment were harvested, threshed, and weighed. Three random samples were then selected to determine the 1000-kernel weight. The grain moisture content was measured using a FOSS-370 near-infrared grain analyzer, and both the actual yield and 1000-kernel weight were adjusted to a standard moisture content of 13%. 

### 2.4. Germination Rate and Mean Germination Time

Germinated seeds are defined as those in which, by the seventh day of germination, the radicle has grown to the full length of the seed and the coleoptile has reached half the length of the seed. The percentage of seeds that germinate each day is recorded. Mean germination time (MGT) is calculated using the formula

MGT = ∑(f × i)/∑ f(1)

where f is the number of newly germinated seeds on day i, and i is the day of germination. A higher MGT value indicates slower germination [26].

### 2.5. Dry and Fresh Weight of Germinating Seeds

On the seventh day of germination, the coleoptile, radicle, and seed remnants were separated. These parts were then dried at 105 °C for 30 min to terminate the enzyme activity, followed by drying at 80 °C until a constant weight was achieved to determine the dry weight.

### 2.6. Water Content Determination

During seed germination, the initial weight of the 30 seeds was measured before the process began. At intervals of 3, 6, 12, 24, 48, and 72 h, seeds were removed from the Petri dishes and blotted dry with a paper towel, and then immediately weighed. The seeds were then returned to the Petri dishes. The seed moisture content was calculated based on the fresh mass using the formula [27]:

Water uptake (%) = [(Fresh mass − Initial mass)/Initial mass] × 100%(2)

where ‘Initial mass’ refers to the initial sample weight; ‘Fresh mass’ refers to the final sample weight at different time periods.

### 2.7. Starch and Soluble Sugars Contents

To extract starch, 1 g of germinated seed powder was mixed with 10 mL of 0.33 mM HCl and heated at 100 °C for 10 min. After extraction, 0.5 mL of 30% (*w*/*v*) ZnSO_4_ was added to the extract to precipitate proteins. Subsequently, 0.5 mL of 30% (*w*/*v*) K_3_ [Fe (CN)_6_] was added to the extract, and the mixture was shaken to ensure thorough mixing, then adjusted to a final volume of 20 mL. After vigorous shaking and filtration, the starch content was analyzed using a polarimeter (WZZ-2B, Shanghai, China) at 20–25 °C, following the method of Xie et al. [28]. For the determination of soluble sugars, 0.1 g of dried germinated seed powder was extracted twice with 80% (*v*/*v*) ethanol at 80 °C. The soluble sugar content was measured using the anthrone method as described by Fares [29].

### 2.8. α-Amylase and β-Amylase Activities

The activities of α-amylase and β-amylase were determined using assay kits (Item No. DFMB-1-Y) provided by Suzhou Keming Biotechnology Co., Ltd. (Suzhou, China). A colorimetric method was employed, where the generation of reducing sugars was detected using the 3,5-dinitrosalicylic acid method. The spectrophotometric analysis was performed at a wavelength of 540 nm. All measurements strictly adhered to the relevant regulations of the “Chinese Pharmacopoeia” and were performed in strict accordance with the manufacturer’s instructions.

### 2.9. ATP Content and Respiration Rate in Seeds

The ATP content was determined using an assay kit (Item No. ATP-1-Y) provided by Suzhou Keming Biotechnology Co., Ltd. (Suzhou, China). The determination was based on the reaction catalyzed by creatine kinase, where creatine and ATP react to produce phosphocreatine. The phosphocreatine content was detected at 700 nm using the phosphomolybdic acid colorimetric method to infer the ATP content. All measurements strictly adhered to the relevant regulations of the “Chinese Pharmacopoeia” and were performed in strict accordance with the manufacturer’s instructions.

Thirty seeds were placed in a small basket and enclosed within a 50 mL wide-mouth jar. To measure the CO_2_ produced during seed respiration, a saturated solution of barium hydroxide [Ba(OH)_2_] was used to absorb the CO_2_. The unreacted barium hydroxide was then quantified by titration with a standard solution of oxalic acid. The formula for calculating the seed respiration rate is:

Seed respiration rate (mg CO_2_ g^−1^ FW h^−1^) = (V_0_ − V_1_)/(mt) (3)

where m is the fresh mass of the seeds (g), t is the measurement time (h), V_0_ is the amount of oxalic acid removed for blank titration (mL), and V_1_ is the amount of oxalic acid removed for sample titration (mL).

### 2.10. Antioxidant Capacity

The malondialdehyde (MDA) content was measured following the method described by Cui et al. [30]. Samples were thoroughly ground in a phosphate buffer solution at pH 7.8 and then centrifuged at 6000 rpm for 10 min. A total of 2 mL of the supernatant was transferred to a graduated test tube, and 1 mL of 0.5% thiobarbituric acid and 3 mL of 5% trichloroacetic acid solution were added. The mixture was heated in a boiling water bath for 10 min and then rapidly cooled. After centrifugation at 6000 rpm for 10 min, the absorbance at 532 nm and 600 nm was measured using a spectrophotometer (UV-2450, Shimadzu Corp., Kyoto, Japan) with distilled water as the blank and transmittance set to 100%. The difference in absorbance between 532 nm and 600 nm was used to calculate the MDA content.

The determination of the ROS release rates was performed using assay kits (Item No. ROS-1-Y) from Suzhou Keming Biotechnology Co., Ltd. (Suzhou, China). The method employs a specific fluorescent probe, 2′,7′-dichlorodihydrofluorescin diacetate. Fluorescence intensity was measured within 10 min at a constant temperature of 37 °C, with an excitation wavelength of 499 nm and an emission wavelength of 521 nm. The rate of change in fluorescence intensity (u) was used as an indicator of the ROS release rate. The activity of peroxidase (POD) was measured using assay kits (Item No. POD-1-Y) from Suzhou Keming Biotechnology Co., Ltd. (Suzhou, China). POD catalyzes the oxidation of H_2_O_2_ by specific substrates, resulting in a characteristic light absorption at 470 nm. The activity of superoxide dismutase (SOD) was determined using assay kits (Item No. SOD-1-Y) from the same company. The xanthine and xanthine oxidase reaction system generates superoxide anion (O_2_^−^), which reduces nitroblue tetrazolium to form a blue formazan with light absorption at 560 nm. SOD scavenges O_2_^−^, thereby inhibiting the formation of formazan. All measurements strictly adhered to the relevant regulations of the “Chinese Pharmacopoeia” and were performed in strict accordance with the manufacturer’s instructions.

### 2.11. K^+^ and Na^+^ Contents

Around 0.1 g of dried germinated seed powder was fully digested at 350 °C with a few drops of 30% H_2_O_2_ (*v*/*v*) and 5 mL of 98% HNO_3_. The resulting solution was then analyzed using inductively coupled plasma-mass spectrometry (ICP-MS; Perkin Elmer, Optima 7300DV, Waltham, MA, USA).

### 2.12. Statistical Analysis

All data were analyzed using one-way analysis of variance (ANOVA) with the SPSS 10.0 software package (SPSS Inc., Chicago, IL, USA). Differences were considered significant between treatments when *p* < 0.05.

## 3. Results

### 3.1. Effects of Exogenous SA Treatment During the Grain-Filling Stage on Grain Weight and Yield in Wheat

**Figure 2:**
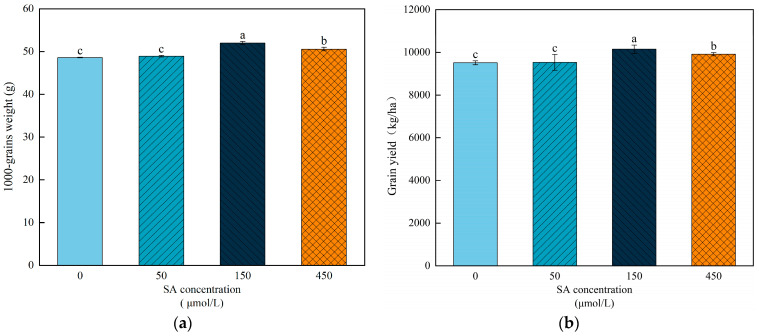
Effects of different SA concentrations on the wheat grain weight and yield. (**a**) Grain weight under different concentrations of SA. (**b**) Grain yield under different concentrations of SA. Each value is the mean ± SE of three biological replicates. The different lowercase letters indicate statistically significant differences at the *p* < 0.05 level.

The effect of the foliar application of SA during the grain-filling stage on the grain weight was in a concentration dependent manner in wheat (Figure 2). The application of 150 μM SA solution significantly increased the grain weight by 6.98% compared to the water treatment, while concentrations of 50 μM and 450 μM resulted in minor changes, with 0.57% and 4.05%, respectively. The response of the wheat grain yield to the SA treatments exhibited a similar trend to grain weight, with the grain yield at 150 μM SA increasing more than at 50 μM and 450 μM (Figure 2). These results indicate that applying appropriate concentrations of SA during the grain-filling stage could significantly increase the grain weight and yield in wheat.

### 3.2. Effects of Exogenous SA Treatment During the Grain Filling in Parental Wheat Plants on Seed Germination Under Salt Stress

**Figure 3:**
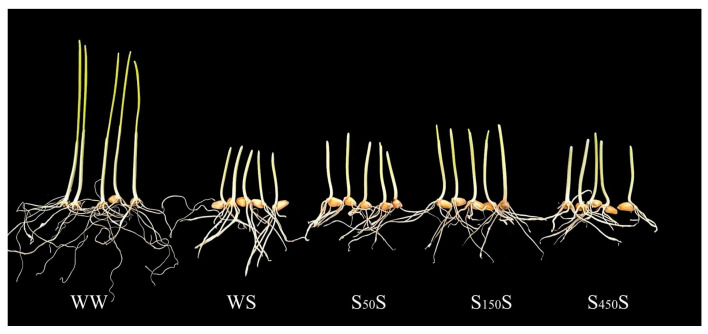
The phenotype of wheat plants at 7 d after germination. Note: WW (water pretreatment + water germination), WS (water pretreatment + NaCl treatment), S_50_S (50 µmol/L SA pretreatment + NaCl treatment), S_150_S (150 µmol/L SA pretreatment + NaCl treatment), S_450_S (450 µmol/L SA pretreatment + NaCl treatment).

As shown in Table 1, the WS treatment caused a significant decrease in the germination rate and an increase in the mean germination time compared to the WW treatment (Table 1). Exogenous SA treatments partially mitigated the salt stress caused the decrease in the germination rate and the increase in the mean germination time. Under the WS treatment, the length and dry mass of the radicle and coleoptile were markedly lower, and the dry mass of the seed residue was significantly higher than that under the WW treatment. However, the seeds of the SA-treated plants showed a higher length and dry mass of the radicle and coleoptile, and a lower dry mass of the seed residue compared to the seeds of the water-treated plants under the salt stress condition. These results suggest that the SA-treated wheat plants during the grain-filling stage could induce salt stress tolerance in their progeny. Among the different concentrations of SA treatment, 150 μmol /L SA (S_150_S) was the most effective spraying concentration during the grain-filling stage to induce transgenerational salt tolerance (Table 1 and Figure 3). Therefore, the underlying mechanisms of SA-induced transgenerational salt tolerance were further explored under the concentration of 150 μmol/L SA.

### 3.3. Effects of Exogenous SA Treatment During the Grain Filling in Parental Wheat Plants on the Imbibition Rate of Progeny Seeds Under Salt Stress

**Figure 4:**
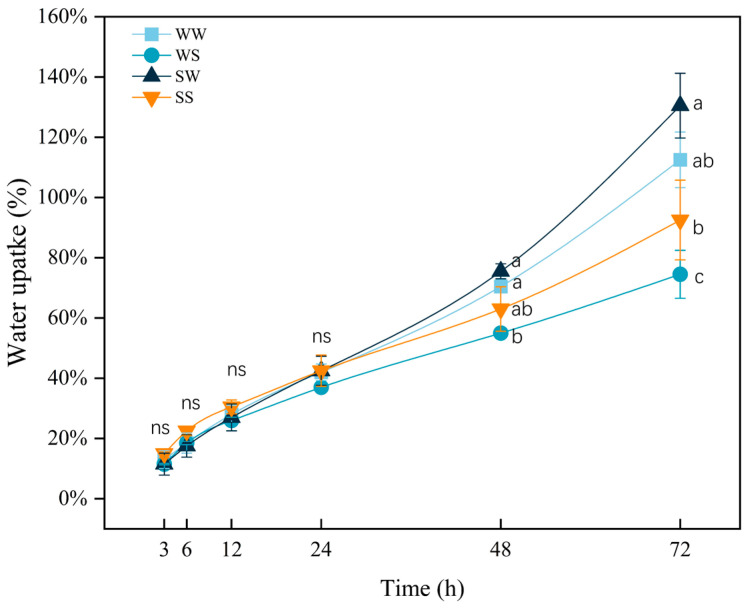
Effects of SA treatment on the water uptake rate of the wheat progeny seeds under salt stress. Note: WW (water pretreatment + water germination), WS (water pretreatment + NaCl treatment), SW (150 µmol/L SA pretreatment + water germination), SS (150 µmol/L SA pretreatment + NaCl treatment). Each value is the mean ± SE of three biological replicates. The different lowercase letters indicate statistically significant differences at the *p* < 0.05 level, “ns” represents no significant difference between the treatments.

As time progressed, the water content of the seeds showed an increasing trend. During the first 3, 6, 12, and 24 h of the experiment, there were no significant differences in the water uptake rates among the different treatment groups (Figure 4). This result indicates that the imbibition of the seeds was not affected by SA and the salt treatment. However, from the 48 h to the 72 h, the water uptake rate of the seeds under salt stress was significantly lower than that of the seeds germinated in water (*p* < 0.05). Notably, under both normal and saline germination conditions, seeds treated with SA showed varying degrees of increased water uptake compared to those treated with water.

### 3.4. Effects of Exogenous SA Treatment During the Grain Filling in Parental Plants on the Degradation of Starch in Progeny Seeds Under Salt Stress

**Figure 5:**
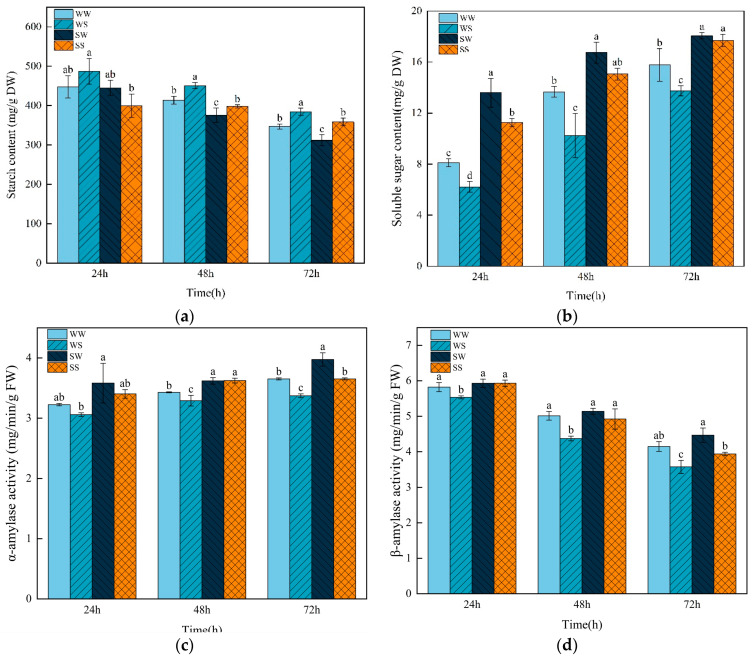
Effects of SA treatment on starch degradation in the wheat progeny seeds under salt stress. (**a**) Starch content; (**b**) soluble sugar content; (**c**) α-amylase activity; (**d**) β-amylase activity. WW (water pretreatment + water germination), WS (water pretreatment + NaCl treatment), SW (150 µmol/L SA pretreatment + water germination), SS (150 µmol/L SA pretreatment + NaCl treatment). Each value is the mean ± SE of three biological replicates. The different lowercase letters indicate statistically significant differences at the *p* < 0.05 level.

Starch content gradually decreased while total soluble sugar content increased along with germination progress in wheat (Figure 5). Salt stress (WS) significantly increased the starch content and decreased the total soluble sugar content at 24 h, 48 h, and 72 h compared to the control treatment (WW). The seeds of plants treated with SA (SW and SS treatment) showed a lower starch content and higher total soluble sugar content than the seeds of the water-treated plants under normal (WW) and salt (WS) germination conditions, respectively.

The activity of α-amylase gradually increased, whereas the activity of β-amylase decreased during seed germination (Figure 5). The WS treatment significantly reduced the activities of both α-amylase and β-amylase in the seeds compared to the WW treatment, but the SS treatment significantly increased the activities of these enzymes related to the WS treatment. Under normal germination conditions, the seeds from the SA-treated plants showed a higher α-amylase activity at 48 h and 72 h than the seeds from the water-treated plants, but there was no significant difference in the activity of β-amylase (Figure 5).

### 3.5. Effects of Exogenous SA Treatment During the Grain Filling in Parental Wheat Plants on ATP Content and Respiration Rate in Progeny Seeds Under Salt Stress

**Figure 6:**
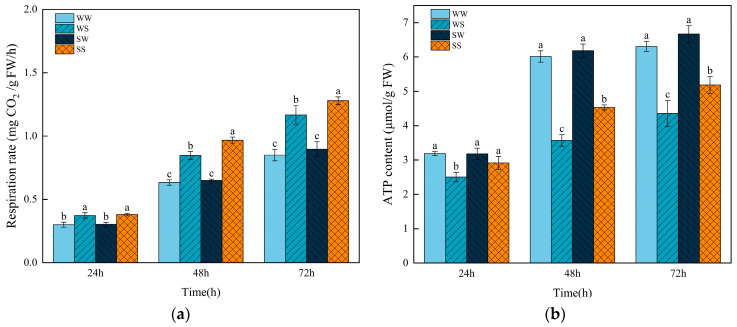
Effects of SA treatment on ATP content and respiration rate in the wheat progeny seeds under salt stress. (**a**) Respiration rate; (**b**) ATP content. WW (water pretreatment + water germination), WS (water pretreatment + NaCl treatment), SW (150 µmol/L SA pretreatment + water germination), SS (150 µmol/L SA pretreatment + NaCl treatment). Each value is the mean ± SE of three biological replicates. The different lowercase letters indicate statistically significant differences at the *p* < 0.05 level.

During the germination of wheat seeds, both the respiration rate and ATP content tended to increase over time (Figure 6). Salt stress significantly increased the respiration rate whereas decreased the ATP content compared to the control treatment. The SS treatment significantly increased the ATP content and respiration rate of the seeds in relation to the WS treatment. Specifically, the ATP content increased by 16.29%, 26.90%, and 19.07% at 24, 48, and 72 h, respectively, while the respiration rate increased by 14.17% and 9.71% at 48 and 72 h, respectively. There were no significant differences in respiration rate and ATP content between the treatment of WW and SW during germination (Figure 6).

### 3.6. Effects of Exogenous SA Treatment During the Grain Filling in Parental Plants on MDA Content and ROS Release Rate in Progeny Seeds Under Salt Stress

**Figure 7:**
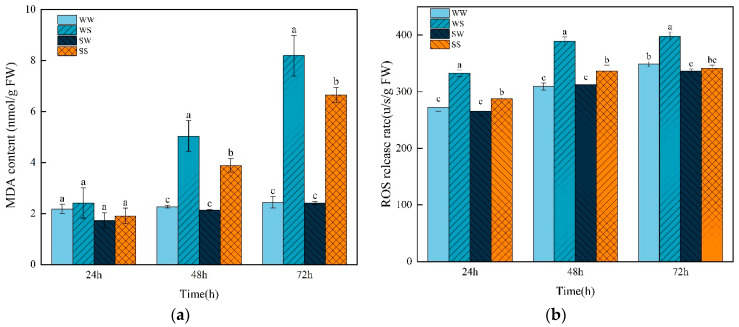
Effects of SA treatment on the MDA content and ROS release rate in the wheat progeny seeds under salt stress. (**a**) MDA content; (**b**) ROS release rate. WW (water pretreatment + water germination), WS (water pretreatment + NaCl treatment), SW (150 µmol/L SA pretreatment + water germination), SS (150 µmol/L SA pretreatment + NaCl treatment). Each value is the mean ± SE of three biological replicates. The different lowercase letters indicate statistically significant differences at the *p* < 0.05 level.

The release rate of ROS and the content of MDA increased during the germination process of wheat seeds. There were no significant differences in the release rate of ROS and the content of MDA between the WW and SW treatment, except in the release rate of ROS at 72 h after germination, where the SW treatment showed significant lower (Figure 7). The WS treatment obviously increased the release rate of ROS and the content of MDA compared with the WW treatment. Compared with the WS treatment, SS treatment significantly reduced the release rate of ROS by 13.49%, 13.57%, and 14.25% at 24, 48, and 72 h after germination, respectively. There was no significant difference in the MDA content between the WS and SS treatment at 24 h after germination, but SS showed a much lower MDA content at 48 h (22.83%) and 72 h (18.85%) after germination.

### 3.7. Effects of Exogenous SA Treatment During the Grain Filling in Parental Plants on the Activities of Antioxidant Enzymes in Progeny Seeds Under Salt Stress

**Figure 8:**
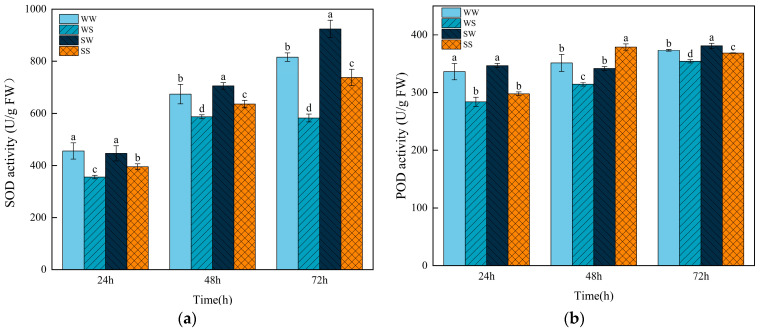
Effects of SA treatment on the SOD and POD activities in the wheat progeny seeds under salt stress. (**a**) SOD activity (**b**): POD activity. WW (water pretreatment + water germination), WS (water pretreatment + NaCl treatment), SW (150 µmol/L SA pretreatment + water germination), SS (150 µmol/L SA pretreatment + NaCl treatment). Each value is the mean ± SE of three biological replicates. The different lowercase letters indicate statistically significant differences at the *p* < 0.05 level.

The activities of SOD and POD increased during the germination of wheat seeds (Figure 8). Compared with the WW treatment, the activities of SOD and POD were significantly inhibited by the WS treatment at 24 h, 48 h, and 72 h (Figure 8). The SS treatment showed significantly higher SOD activity at 24 h (11.10%), 48 h (8.21%), and 72 h (26.59%), and a higher POD activity at 48 h (20.60%) than those in the WS treatment. The activity of SOD was much higher in the SW treatment at 48 h and 72 h after germination compared to the WW treatment. In addition, the SW treatment markedly increased the POD activity at 72 h after treatment relative to the WW treatment (Figure 8).

### 3.8. Effects of Exogenous SA Treatment During the Grain Filling in Parental Plants on Sodium (Na^+^) and Potassium (K^+^) Ion Contents in Progeny Seeds Under Salt Stress

**Figure 9:**
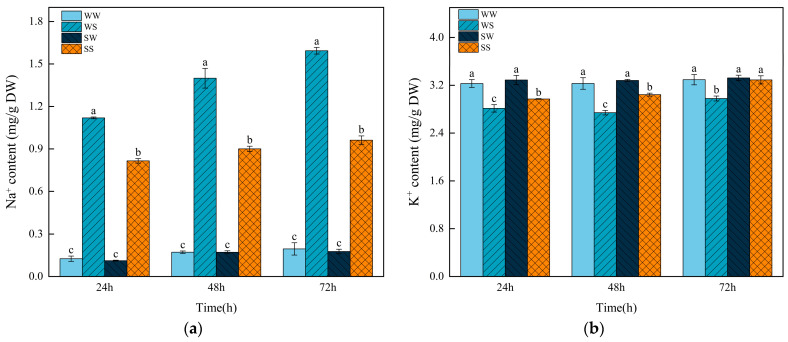
Effects of SA treatment on the Na^+^ and K^+^ contents in the wheat progeny seeds under salt stress. (**a**) Na^+^ content; (**b**) K^+^ content. WW (water pretreatment + water germination), WS (water pretreatment + NaCl treatment), SW (150 µmol/L SA pretreatment + water germination), SS (150 µmol/L SA pretreatment + NaCl treatment). Each value is the mean ± SE of three biological replicates. The different lowercase letters indicate statistically significant differences at the *p* < 0.05 level.

The WS treatment strikingly increased the Na^+^ content while it decreased the K^+^ content at 24 h, 48 h, and 72 h after germination relative to the WW treatment (Figure 9). Compared to the WS treatment, the SS treatment significantly reduced the Na^+^ content by 27.13%, 35.65%, and 39.66% and increased the K^+^ content by 5.58%, 11.00%, and 10.48% at 24, 48, and 72 h after germination, respectively. There were no significant differences in the contents of Na^+^ and K^+^ between the treatments of WW and SW.

## 4. Discussion

Salicylic acid is a multifunctional signal substance in plants that plays an important role in regulating plant growth and development and stress responses [31]. Previous studies have shown that the foliar application of SA at early or later stages can significantly increase the grain yield of wheat [32,33]. In this study, the application of exogenous SA during the grain-filling stage significantly increased the grain yield of wheat, which was mainly due to the increase in grain weight (Figure 2). It has been documented that applying appropriate concentrations of SA could enhance the nitrogen use efficiency, leaf chlorophyll content, and photosynthetic efficiency in plants [34,35]. These results suggest that SA treatment increased the grain weight and yield of wheat by improving photosynthetic efficiency. In addition, the effects of SA on grain weight and yield were in a dose-dependent manner, and 150 μM SA was more effective than other concentrations in this study. This finding is consistent with the results of previous studies that reported that the application concentration is an important factor affecting the regulatory effects of SA on senescence and stress tolerance in plants [36].

Although the important role of endogenous SA in biotic stress induced trans-generational stress tolerance has been recorded [37,38], there are few studies on the effect of exogenous SA on offspring tolerance in plants. Numerous studies have shown that exogenous SA treatment could effectively increase the contemporary salt stress tolerance in many species including wheat [17,39,40]. In the present study, the exogenous application of SA during the grain-filling stage significantly enhanced the salt tolerance of the progeny seeds in wheat, as demonstrated by the higher germination rate, length and dry mass of the coleoptile and radicle, and a lower mean germination time compared to the seeds of the water-treated plants under the salt germination condition (Table 1). These results were consistent with findings that a SA soaking treatment promotes seed germination under salinity stress in crops including barely, rice, and wheat [20,41]. However, to our knowledge, this is the first report that the application of SA can induce trans-generational tolerance to abiotic stress in plants.

The germination process can be considered in terms of three sequential steps: imbibition, reserve mobilization, and radicle and coleoptile elongation [42]. The process of wheat seed imbibition is usually completed within 12 h [43]. In this study, SA and salt stress treatment did not affect the increase in fresh weight in the wheat seeds with 24 h after germination (Figure 4), indicating that the shorter germination time of seeds from the SA-treated plants under salt stress was not caused by enhanced seed imbibition. However, the seeds of SA-treated plants showed a greater increase in fresh weight at 48 h and 72 h after germination compared to the seeds of water-treated plants under salt stress (Figure 4). This result was associated with the higher soluble sugar content in the seeds of SA-treated plants than the seeds of water-treated plants under salt stress (Figure 4 and Figure 5). Soluble sugar plays a crucial role in regulating osmotic balance in plants [44]. These results suggest that the seeds from SA-treated plants could accumulate more osmoregulatory substance to alleviate osmotic damage caused by salt stress.

Starch stored in the endosperm is hydrolyzed into soluble sugars, which can be further converted into energy and intermediate metabolites for seed germination. Previous studies have shown that salt stress inhibited the mobilizing of starch by reducing hydrolase activity, thereby delaying seed germination [10]. A recent study found that SA soaking treatment significantly increased the activity of α-amylase and the content of soluble sugar in rice seeds under salt stress [20]. In the present study, the seeds from the SA-treated plants showed higher hydrolase (α-amylase and β-amylase) activities and lower starch content compared to the seeds from the water-treated plants under salt stress, indicating that the seeds from SA-treated plants could better utilize starch in the endosperm, thus maintaining a higher soluble sugar content and water absorption rate under salt stress. However, high concentrations of SA inhibited the activity of amylase and seed germination [45]. From this perspective, the treatment method of spraying SA during the grain-filling stage to promote seed salt tolerance might be safer compared to SA soaking.

In this study, in addition to inhibiting starch hydrolysis, salt stress also significantly reduced the ATP content in wheat and increased the respiration rate of the wheat seeds. This result is consistent with previous reports that stated that salt stress increases seed respiration [46,47,48]. The increasing respiration rate was beneficial for producing more energy in response to stresses. However, the excessive energy consumption under salt stress might be the reason for the decrease in ATP content in the wheat seeds in the present study. Zapata [49] and others found that the respiration rates of lettuce, melon, and pepper seeds increased with rising salinity, but the salinity of spinach did not increase. This result suggests that the effect of salt on seed respiration rates may be related to the species’ sensitivity to salt stress. Under salt stress, seeds from the SA-treated plants exhibited significantly higher respiration rates and ATP content compared to those from the water-treated plants (Figure 6), indicating that seeds from the SA-treated plants were able to maintain sufficient energy levels for seed germination under salt stress.

In addition to causing osmotic damage, salinity also induces an imbalance of the inner cellular ions, thus leading to ion toxicity in plant cells [50]. In this study, salt stress (NaCl treatment) dramatically increased the concentration of Na^+^ while reducing the concentration of K^+^ in the wheat seeds (Figure 9). These results are consistent with previous findings that plants absorb excessive Na^+^ as the amount of Na^+^ in the growth medium increases, resulting in an increase in K^+^ efflux from cells [51]. However, SA treatment during the grain-filling stage significantly alleviated the changes in Na^+^ and K^+^ concentrations compared to the water treatment under the salt germination condition (Figure 9). This result suggests that SA could improve the wheat seed salt tolerance through maintaining ion homeostasis. Liu [20] et al. (2022) found that a SA soaking treatment upregulated the expression levels of *OsHKTs* in rice seeds, which promoted Na^+^ efflux, thus maintaining ion balance under salt stress.

Reactive oxygen species are highly reactive and toxic; their overaccumulation can lead to the oxidative destruction of nucleic acids, proteins, lipids, etc. [52]. MDA is the product of the membrane lipid oxidation reaction and is often used to reflect the degree of cellular peroxidation damage under stresses [6]. In this study, salt stress significantly increased the rate of ROS production and MDA content in the wheat seeds, whereas SA pretreatment effectively relieved these changes (Figure 7). Similar observations have been recorded in rice, wheat, and *Arabidopsis thalianan*, where exogenous SA enhanced the salt tolerance by activating antioxidant systems under salt stress [53,54,55,56]. SOD and POD are two important enzymes in the plant antioxidant system [57]. Spraying SA during the grain-filling stage significantly increased the activities of SOD and POD in the progeny seeds under salt stress in this study (Figure 8), indicating that SOD and POD are involved in the enhancement of antioxidant capacity in the seeds of the SA-treated plants under salt stress. In addition, exogenous SA treatment increased the contents of glutathione and ascorbate in salt-stressed wheat seedlings [58]. These results indicate that enhancing the antioxidant capacity is an important mechanism by which SA enhances plant tolerance to salt stress.

Previous studies have found that exogenous SA treatment could improve the salt tolerance of seeds and plants in many crops in the current generation [19,20,22]. In this study, we found that the application of SA during the grain-filling stage in wheat plants could effectively improve the progeny seeds’ salt tolerance by maintaining osmotic, ion, and redox homeostasis in cells under salt stress. Spraying SA during the grain-filling stage may have an impact on the formation of wheat seeds such as the content of endogenous SA in seeds, which in turn affects the salt tolerance of seeds. It has been documented that SA and its analogue induced long-term priming and transcriptional memory in plant immunity through the DNA methylation pathway [59]. For example, BTH (an SA analogue) significantly upregulated the expression level of *AMY1* (*amylase 1*) through DNA methylation in *Arabidopsis* [60]. Consistent with this result, in the present study, the seeds from the SA-treated plants exhibited higher activities of α-amylase and β-amylase under salt stress compared to the seeds from the water-treated plants (Figure 5). Thus, further investigation is needed to determine whether epigenetic modifications mediate SA-induced salt tolerance in wheat seeds across generations, as recorded in biotic stress [61]. Based on the results of this study, SA application may improve seed yield. The added expense of SA treatment for general agricultural production may be greater than the value of the yield increase. However, the value-added trait of seeds epigenetically primed with enhanced stress tolerance may be worth the expense of SA application in the field by growers producing seeds to sell to farmers for the next growing season.

## Figures and Tables

**Table 1:** Effects of different SA concentrations on the germination and growth of the wheat progeny seeds under salt stress.

Treatment	Germination Rate (%)	Mean Germination Time (Day)	Length (cm)		Dry Weight (mg seed^−1^)		
			Coleoptile	Radicle	Seed Residue	Coleoptile	Radicle
WW	98.00 ± 0.02 a	3.27 ± 0.04 d	9.88 ± 0.13 a	7.76 ± 0.13 a	22.13 ± 0.46 d	7.67 ± 0.24 a	3.98 ± 0.19 a
WS	64.00 ± 0.03 d	3.72 ± 0.05 a	4.72 ± 0.09 e	4.37 ± 0.07 d	30.84 ± 0.41 a	4.37 ± 0.43 d	1.87 ± 0.28 c
S50S	73.33 ± 0.04 c	3.63 ± 0.07 ab	5.18 ± 0.12 d	4.59 ± 0.09 d	28.27 ± 0.83 b	5.28 ± 0.40 c	2.42 ± 0.07 b
S150S	80.00 ± 0.02 b	3.50 ± 0.08 c	5.78 ± 0.06 b	5.43 ± 0.12 b	24.60 ± 0.53 c	6.22 ± 0.10 b	2.56 ± 0.10 b
S450S	76.67 ± 0.03 bc	3.55 ± 0.06 bc	5.47 ± 0.14 c	5.03 ± 0.26 c	27.75 ± 0.59 b	5.58 ± 0.13 c	2.44 ± 0.12 b

Note: WW (water pretreatment + water germination), WS (water pretreatment + NaCl treatment), S_50_S (50 µmol/L SA pretreatment + NaCl treatment), S_150_S (150 µmol/L SA pretreatment + NaCl treatment), S_450_S (450 µmol/L SA pretreatment + NaCl treatment). Each value is the mean ± SE of three biological replicates. The different lowercase letters indicate statistically significant differences at the *p* < 0.05 level.

## Data Availability

Data are contained within the article.
